# Insights into Photocatalytic Degradation Pathways and Mechanism of Tetracycline by an Efficient Z-Scheme NiFe-LDH/CTF-1 Heterojunction

**DOI:** 10.3390/nano12234111

**Published:** 2022-11-22

**Authors:** Jinpeng Zhang, Xiaoping Chen, Qiaoshan Chen, Yunhui He, Min Pan, Guocheng Huang, Jinhong Bi

**Affiliations:** 1 School of Environmental Science and Engineering, Fuzhou University, Fuzhou 350108, China; 2 Fujian College Association Instrumental Analysis Center of Fuzhou University, Fuzhou 350108, China; 3 Department of Applied Science, School of Science and Technology, Hong Kong Metropolitan University, Ho Man Tin, Kowloon, Hong Kong SAR, China

**Keywords:** layered double hydroxide, photocatalysis, antibiotic

## Abstract

Photocatalysis offers a sustainable approach for recalcitrant organic pollutants degradation, yet it is still challenging to seek robust photocatalysts for application purposes. Herein, a novel NiFe layered double hydroxide (LDH)/covalent triazine framework (CTF-1) Z-scheme heterojunction photocatalyst was rationally designed for antibiotics degradation under visible light irradiation. The NiFe-LDH/CTF-1 nanocomposites were readily obtained via in situ loading of NiFe-LDH on CTF-1 through covalent linking. The abundant coupling interfaces between two semiconductor counterparts lay the foundation for the formation of Z-scheme heterostructure, thereby effectively promoting the transfer of photogenerated electrons, inhibiting the recombination of carriers, as well as conferring the nanocomposites with stronger redox ability. Consequently, the optimal photocatalytic activity of the LDH/CTF heterojunction was significantly boosted for the degradation of a typical antibiotic, tetracycline (TC). Additionally, the photodegradation process and the mineralization of TC were further elucidated. These results envision that the LDH/CTF-1 can be a viable photocatalyst for long-term and sustainable wastewater treatment.

## 1. Introduction

Antibiotics have become widely utilized in treating infections for humans and veterinary, as well as in agriculture for crop improvement [1]. Up to now, Tetracycline (TC) has become one of the most common antibiotics for use and production [2]. Nonetheless, with the recent increase in antibiotic use, the discharge of antibiotic residues into aquatic habitats is unavoidable, leading to refractory contamination sources [3]. Antibiotics and their degradation products may have severe implications for the environment, for example, antibiotics could enhance bacterial resistance or damage key production cycles of crops and animals [4]. As a result, there is a pressing need for sustainable and environmentally friendly technology to address the related concerns.

Recently, semiconductor-based photocatalysis emerges as a compelling and viable strategy for solving the problem of recalcitrant organic pollution because of the combined merits of green, low-cost, recyclable, and using renewable solar energy [5,6]. However, the application of photocatalysts in organic pollutant degradation is largely plagued by the undesirable recombination of photo-generated electrons and sluggish transfer of the charge carriers [7]. More recently, interfacial engineering through the construction of Z-scheme binary photocatalysts has been widely demonstrated as a valid approach to tackle these issues. The Z-scheme photocatalysts are typically built upon two photocatalysts with staggered band structure and abundant coupling interfaces. The Z-scheme electron transfer pathway is intrinsically beneficial for the efficient migration of charge carriers [8]. To date, various Z-scheme heterostructure photocatalysts, such as Bi-based [9,10], metal oxide-based [11], and metal sulfide-based [12] heterojunctions, have attracted tremendous interest in antibiotic degradation. Despite substantial advances, classic Z-scheme heterostructure photocatalysts are limited in their practical applications due to their aligned band topologies, interfacial contact area, and substrate transmission during photocatalytic processes, resulting in low photocatalytic efficiency [13]. Lately, a combination of inorganic and organic semiconductors has been proposed as a feasible strategy to improve the photocatalytic efficiency of photocatalysts [14,15,16].

The molecular formula of layered double hydroxides of non-precious metals (LDHs) is [M^2+^_(1−x)_M^3+^_x_(OH)_2_]^x+^[A^n−^_x/n_]·mH_2_O, which is a group of inorganic compounds similar to hydrotalcite and is used to balance the residual positive charge on the LDH, where M^2+^ is a divalent metal ion, M^3+^ is a trivalent metal ion, and A^n−^ is an organic or inorganic anion [17,18]. LDHs have become attractive candidates for cocatalysts because of their unique properties, including a considerable specific surface area, superior ion exchange capabilities, an orderly layered structure, and strong maneuverability. Additionally, a variety of cation compositions and layer-intercalated anion choices are available, allowing for fine interfacial tailoring and changeable modulation of the band gap of LDHs via coupling with other materials. Pure LDH, on the other hand, has an inadequate photocatalytic performance due to fast charge recombination following irradiation [17], and thus modifications were commonly made.

In the past decade, covalent triazine-based frameworks (CTFs) have received tremendous attention as newly developing organic carbon material for photocatalytic application. Due to the unique features such as tailorable band gap energies, customizable layered structure, and high thermal/chemical stability [19], CTFs exhibited valuable potential in visible-light-driven photocatalytic environmental remediation [20,21]. It has been demonstrated that the triazine-based-conjugated framework of CTFs is beneficial for broadening their photoresponsive range, and stacking layers structure in CTFs can speed up photoinduced carrier transfer [22]. It is not known yet how CTFs will perform in a Z-scheme heterostructure photocatalytic system for organic pollutant degradation.

The hypothesis that the Z-scheme heterostructure can increase the interfacial contact area between two semiconductors allows for better visible light absorption and photogenerated electron and hole (e^−^/h^+^) separation and transfer kinetics. For the sake of boosting photocatalytic behavior, the forceful electronic coupling effects at the boundary of heterostructure can be exploited to change the interaction between components and vary their electronic structures.

Herein, we developed a Z-scheme heterostructure LDH/CTF-1 via the LDH nanosheets covering the CTF-1 surface and choose a typical antibiotic TC as a targeted contaminant to explore the photocatalytic ability of LDH/CTF-1 under visible light irradiation in this work. Compared with a single semiconductor counterpart, the LDH/CTF-1 photocatalyst can form a Z-scheme heterojunction between LDH and CTF-1, so that photogenerated e^−^/h^+^ couples will separate significantly. As a result, the as-prepared photocatalyst has good TC removal ability and stability, especially when the mass ratio LDH/CTF-1 = 40%, paving the door for the development of further Z-scheme photocatalysts.

## 2. Materials and Methods

### 2.1. Preparation of CTF-1

CTF-1 was synthesized based on our previous work [23]. Firstly, 5.12 g 1,4-dicyanobenzene was added to a beaker and slowly injected with 40 mL Trifluoromethane acid at 273 K. The obtained solution was stirred at 303 K until it solidified and then left to stand for 3 days. Dichloromethane (2 × 80 mL) and NH_3_·H_2_O (3 × 200 mL) were used to wash the obtained solid and then redispersed in NH_3_·H_2_O (200 mL). After centrifuging with ultrapure water and methanol, the obtained solid was extracted respectively by methanol and Dichloromethane using Soxhlet extraction. The precipitate was dried in a vacuum oven at 353 K for 12 h. Then, the pure CTF-1 was obtained after grinding.

### 2.2. Preparation of LDH/CTF-1

NiFe-LDH/CTF-1 nanocomposites were synthesized by a reported method with minor modifications [24]. In short, the as-prepared CTF-1, Ni(NO_3_)_2_·6H_2_O (0.48 mmol), and Fe (NO_3_)_3_·9H_2_O (0.24 mmol) were firstly dispersed in 100 mL ultrapure water with ultrasonic treatment for 30 min and stirred for 2 h. NH_4_F (54 mM) was then dissolved in the above solution. After that, a 60 mL aqueous solution containing 0.72 mmol NaOH and 1.8 mmol Na_2_CO_3_ was added dropwise under vigorous stirring at room temperature. After agitating for 5 h, the suspension will be aged for 1 d, washed with water and ethanol several times to make the pH reach 7, and collected by centrifuging. Finally, an earthy yellow powder was obtained across freezing-drying for 12 h. The initial mass ratio of CTF-1 to NiFe-LDH was regulated at 20%, 40%, 50%, and 60%; meanwhile, the corresponding products were denoted as LDH/CTF-1-20%, -40%, -50%, and -60%, respectively. As a comparison, we used the same method without CTF-1 to obtain the pure NiFe-LDH, which was called LDH. A mechanical physical mixing LDH and CTF-1 sample (Mix) was also prepared as a comparison, with the mass ratio LDH/CTF-1 = 40%.

### 2.3. Photocatalytic Activity Tests

The photocatalytic activities of the nanocomposites were evaluated by the degradation of TC under visible light irradiation (λ > 420 nm). Visible illumination was obtained by a 300 W Xe lamp (PLS-SXE300C, Beijing, China) equipped with a 420 nm cutoff filter. Typically, 20 mg of the sample were dispersed into a 100 mL of 40 mg L^−1^ TC aqueous solution. Before irradiation, the suspensions were fiercely magnetically stirred for 120 min in the dark to reach the adsorption–desorption equilibrium. Then, visible light irradiation was carried out. At given reaction time intervals, 2 mL of the reaction suspensions were taken out and filtered through a 0.22 μm membrane to remove the photocatalyst powder, and the concentration of TC was monitored by a UV-vis spectrophotometer (GEN10S UV-vis, Boston, MA, USA) by checking its characteristic absorbance at 357 nm.

The degradation efficiency (*DE*, %) was calculated by the following equation:

$$DE\;\left(\%\right)=\frac{C_{0}-C_{t}}{C_{0}}$$

where *C*_0_ (mg L^−1^) is the initial concentration of TC, and *C_t_* is the concentration of TC after the irradiation time of t.

For trapping experiments, BQ (1 mM), IPA (1 mM), and EDTA-2Na (1 mM) were used as scavengers of superoxide radicals (·O_2_^−^), hydroxide radicals (·OH), and photogenerated holes (h^+^), respectively. The concentration of TC was measured by Shimadzu Prominence LC-20A high-performance liquid chromatograph (HPLC). The total organic carbon (TOC) assays were conducted on a Shimadzu TOC-L CPH analyzer (Kyoto, Japan).

### 2.4. Characterization and Performance Measurement

All details are shown in the supporting information, including material and chemical characterization, and identification of the degradation intermediates.

## 3. Results and Discussion

### 3.1. Crystal Structure, Morphology, and Physicochemical Properties

**Figure 1:**
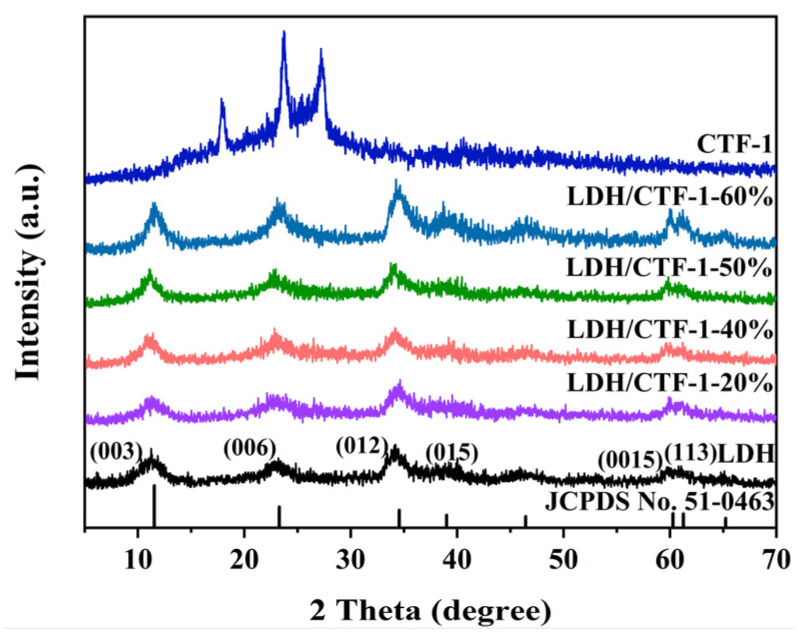
XRD patterns of pure LDH, CTF-1, and the LDH/CTF-1 nanocomposites.

Powder X-ray diffraction (PXRD) spectroscopy was used to analyze the crystal structure of the synthesized samples, which was compared in Figure 1. The (003), (006), (012), (015), (018), (110), and (113) lattice planes, proving the hydrotalcite-type NiFe-LDH structure, are ascribed to the reflections at 11.3°, 23.1°, 34.2°, 39.0°, 46.4°, 60.1°, and 61.1° for pure NiFe-LDH (JCPDS No. 51-0463) [25]. Diffraction peaks for pure CTF-1 were found at 17.8°, 23.6°, and 27.3°, respectively. The peak at 27.3 ° was associated with the accumulation of layered aromatic structure [26], whereas the other two peaks were related to oligomer formation [27]. It should be highlighted that no CTF-1 peaks can be seen in the LDH/CTF-1 nanocomposites, presumably because the signal of CTF-1 is hidden by the development of LDH nanosheets on the surface of CTF-1. However, with the increased amount of CTF-1, the CTF-1/LDH-25% exhibits all the characteristic diffraction peaks of CTF-1, indicating the co-existence of LDH and CTF-1 (Figure S1).

**Figure 2:**
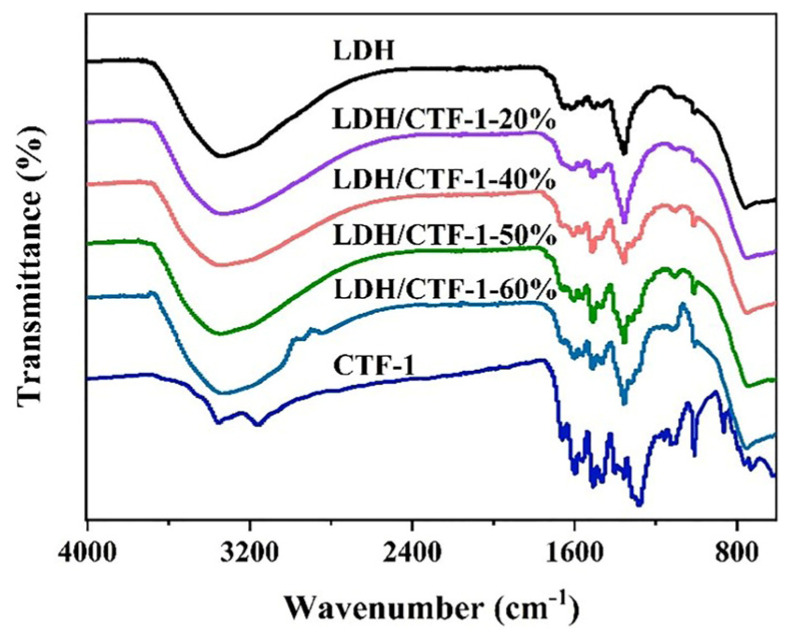
FT-IR spectra of pure LDH, CTF-1, and the LDH/CTF-1 nanocomposites.

Figure 2 shows the Fourier transform infrared (FT-IR) spectra of LDH, CTF-1, and LDH/CTF-1 nanocomposites. The strong band at 1563 cm^−1^ in pure LDH could be caused by bending vibrations of CO_3_^2−^ ions intercalated in the lamellar structure [28], whereas the other bands below 800 cm^−1^ are attributed to the translational modes of metal–oxygen (Ni-O and Fe-O) and metal–oxygen–metal (Ni-O-Fe) bands [29,30]. In CTF-1, the typical adsorption peaks at 1508 and 1357 cm^−1^ are attributable to conventional triazine ring stretching modes [31]. The CTF-1 diffraction peaks steadily intensify as CTF-1 content increases, at the expense of LDH peaks. Meanwhile, large bands around 3359 cm^−1^ and 1623 cm^−1^ are also seen, ascribing to the hydroxyl stretching mode corresponding to metal hydroxyl groups coupled with hydrogen-bonded interlayer water molecules and the hydroxyl deformation mode of water [28,32].

**Figure 3:**
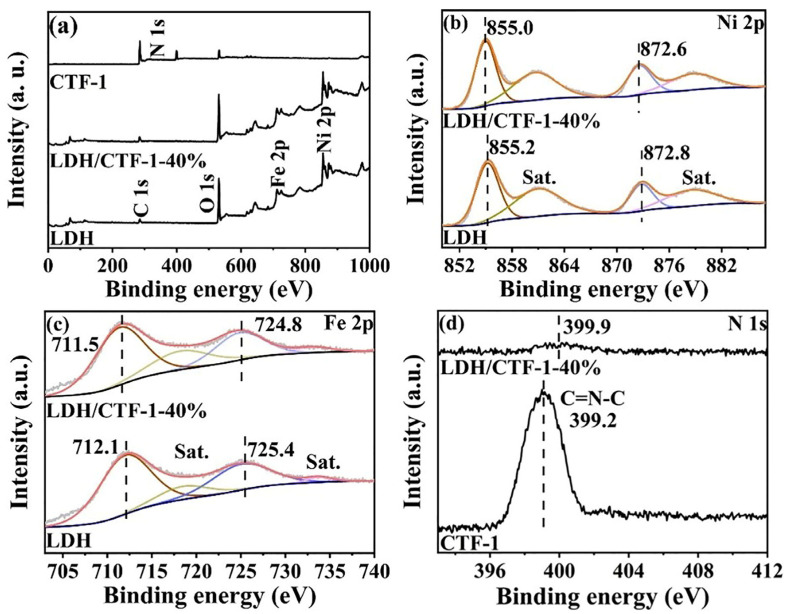
(**a**) XPS survey spectra, high-resolution X-ray photoelectron spectra in (**b**) Ni 2p; (**c**) Fe 2p; and (**d**) C 1s regions of LDH, CTF-1, and LDH/CTF-1-40%.

X-ray photoelectron spectroscopy (XPS) analysis was used to determine the surface chemical states and composition of catalysts. The survey spectra of pure LDH, CTF-1, and LDH/CTF-1-40% samples are shown in Figure 3a. LDH/CTF-1-40% exhibits the peaks corresponding to C, N, O, Fe, and Ni elements, indicating the existence of LDH/CTF-1 nanocomposites. The high-resolution areas of Ni 2p for the as-synthesized LDH and LDH/CTF-1-40% samples are compared in Figure 3b. Two spin–orbit doublets, Ni 2p_3/2_ and 2p_1/2_, appear at the binding energy of 855.2 and 872.8 eV and two associated shake-up satellites (Sat.) of LDH may be fitted to four peaks, implying that the nickel element in LDH is in the divalent form [30]. In addition, the high-resolution Fe 2p spectra of LDH (Figure 3c) show a set of Fe 2p_3/2_ and 2p_1/2_ peaks at 712.1 and 725.4 eV, as well as two satellite peaks (Sat.) attributable to the existence of Fe^3+^ species in LDH [33]. The peaks at 399.2 eV in the N 1s spectra of CTF-1 (Figure 3d) correspond to sp^2^-hybridized aromatic nitrogen (pyridinic N) in the triazine ring [34]. Furthermore, the binding energies for the Ni 2p and Fe 2p areas in the LDH/CTF-1-40% nanocomposite are 0.2 and 0.6 eV lower than those of pure LDH, respectively. The peak of N 1s, on the other hand, shifts positively by 0.7 eV. This event shows that photo-excited charge carriers can help LDH and CTF-1 communicate.

**Figure 4:**
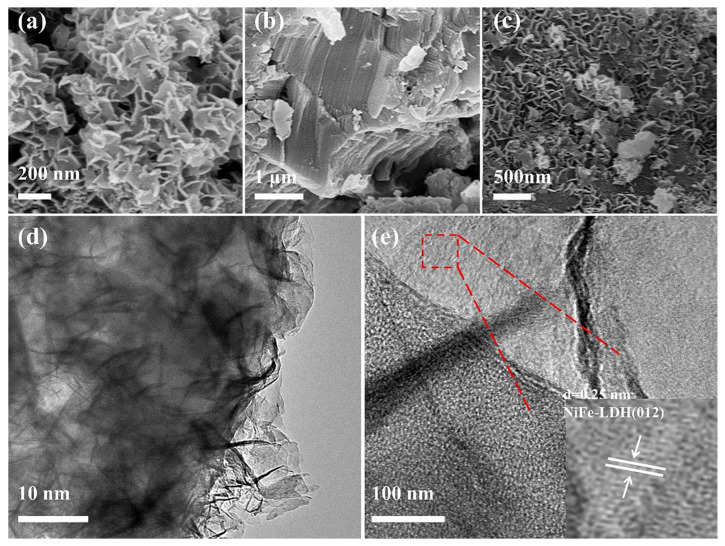
HRSEM images of (**a**) LDH; (**b**) CTF-1; (**c**) LDH/CTF-1-40%.TEM images of (**d**,**e**) LDH/CTF-1-40%.

The morphology of the nanocomposite was investigated by ultra-high-resolution scanning electron microscopy (HRSEM) and transmission electron microscopy (TEM). The pure LDH (Figure 4a) is made up of several small nanosheets with a diameter of 100–300 nm that tend to agglomerate into bigger aggregates of several micrometers. CTF-1 had a tiered stacking structure when it was in its natural state (Figure 4b). The HRSEM and TEM photos (Figure 4c,d) of LDH/CTF1-40% samples clearly show the uniform NiFe-LDH nanosheets dispersed on the CTF-1 surface, albeit a few loose nanosheets are occasionally visible. Closer observation reveals that average thickness of the ultrathin nanosheets is less than 10 nm. When photocatalysis occurs, the widespread distribution of LDH on the surface of CTF-1 could greatly contribute to quantum efficiency because of the tight binding at the interface [35]. In addition, the visible equally lattice fringes with a spacing of 0.25 nm can be seen in Figure 4e, which corresponds to the (012) plane of the pristine LDH while CTF-1 shows an amorphous state [24,25], which further confirms the coexistence of LDH and CTF-1. The EDS mapping results (Figure S2) indicate that C, N, O, Ni, and Fe are uniformly distributed in LDH/CTF-1, further confirming the formation of LDH/CTF-1 heterostructure.

**Figure 5:**
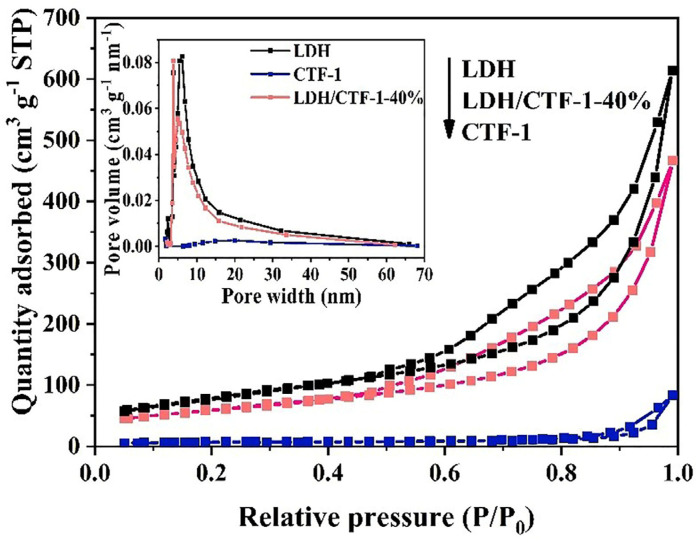
Nitrogen adsorption–desorption isotherms and relevant pore size distribution curves (inset) of LDH, CTF-1, and the LDH/CTF-1-40% samples.

The Brunauer-Emmett-Teller (BET) and pore size distributions are calculated by N_2_ adsorption–desorption isotherms. All isotherms and hysteresis loops were matched with conventional IV isotherms (P/P_0_ > 0.6) and H3-type (Figure 5), showing that mesoporous materials with wedge-shaped holes generated by flaky loose buildup have characteristic adsorption lines [30,36], which correspond to the results observed by SEM. As illustrated in Figure 5, the pore size distribution curves show that the pore size distributions of LDH and the LDH/CTF-1-40% samples are similar in the region of 2 nm to 70 nm. Due to the aggregation of LDH nanosheets and the self-assembly of LDH in CTF-1/LDH onto CTF-1, we can see an obvious peak at 2–10 nm. These results are consistent with the above examination of the isotherms. The BET surface area and pore volume (Table 1) of LDH/CTF-1-40% (206.6 m^2^ g^−1^ and 0.72 cm^3^ g^−1^ nm^−1^, respectively) are lower than those of pristine LDH (281.3 m^2^ g^−1^ and 0.95 cm^3^ g^−1^ nm^−1^, respectively). The stacking of LDH on the CTF-1 covered part of their stack pores with LDH nanosheets, which are responsible for their morphological properties, result in a 40% reduction in surface area compared to a single LDH.

### 3.2. Optical and Photoelectrochemical Properties

**Figure 6:**
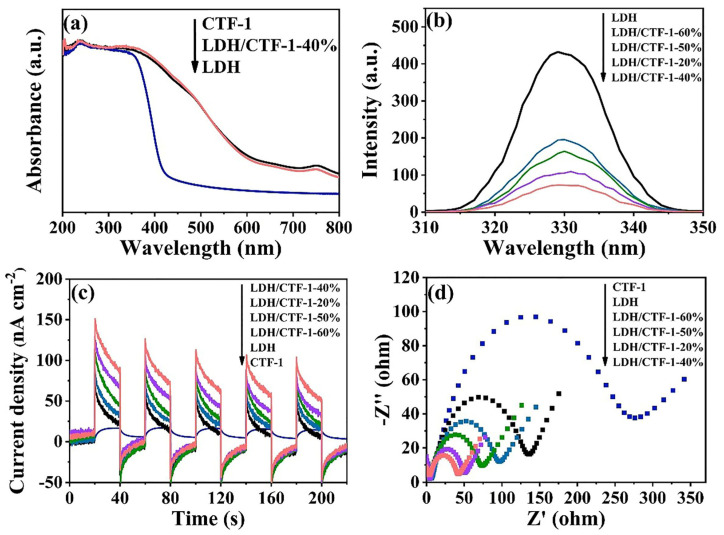
(**a**) UV-vis DRS; (**b**) PL spectra; (**c**) photocurrent responses; (**d**) EIS Nyquist plots for the pure LDH, CTF-1, and the LDH/CTF-1 nanocomposites.

Use the UV–vis diffuse reflectance spectra (UV-vis DRS) to evaluate the optical characteristics of LDH, CTF-1, and the LDH/CTF-1 samples (Figure 6a). The optical semiconductor absorption morphologies of the LDH/CTF-1-40% and bare LDH samples were identical, showing that the intrinsic semiconducting nature of LDH was preserved following the addition of CTF-1. The intrinsic absorption of LDH, which involves the ligand metal and metal–metal charge transfer, produces a band at 200–318 nm [37,38], and another absorption peak around 750 nm is associated with d-d transitions of Ni^2+^ ions in LDH [29]. After the addition of CTF-1, the maximum absorption edge of LDH/CTF-1-40% showed a small blue shift. The strong interfacial interactions between CTF-1 and LDH are demonstrated, which play an important part in improving the charge transfer to enhance the photocatalytic activity. Moreover, for confirming the respective band gap, the plots of the specific absorption band edge using a transformed Kubelka–Munk function to calculate [39,40] (Figure S3), and the corresponding band gap (Eg) energy of pure LDH, CTF-1, and LDH/CTF-1-40% samples is calculated to be 2.34, 2.94, and 2.40 eV, respectively.

When compared to LDH, the photoluminescence (PL) intensity of LDH/CTF-1 heterojunction drops dramatically, as seen in Figure 6b, which means that the introduction of CTF-1 could effectively suppress the photocarriers’ recombination of the LDH, benefitting the separation of electron–hole pairs. This may be due to the band structure matching between LDH and CTF-1, so the introduction of CTF-1 can effectively inhibit the optical carrier recombination of LDH, thus improving the carrier transmission efficiency.

The transient photocurrent responses and electrochemical impedance spectroscopy (EIS) analyses were used to further examine the charge migration and recombination properties. The photocurrent of the LDH/CTF-1-40% nanocomposite is substantially higher than that of bare LDH, CTF-1, and the other ratio LDH/CTF-1 nanocomposites, as shown in Figure 6c, showing that the LDH/CTF-1-40% nanocomposite has the fastest charge separation rate. This could be due to the synergistic impact of the LDH/CTF-1 heterojunction, which considerably improves the separation of photo-generated e^−^/h^+^ pairs, prevents charge recombination, and extends the lifespan of charge carriers, enhancing photocurrent density. In addition, EIS analysis results are similar. Figure 6d exhibits the EIS difference of LDH, CTF-1, and LDH/CTF-1 nanocomposite. We can clearly see that the relative size of the arc radius is CTF-1 > LDH > LDH/CTF-1-60% > LDH/CTF-1-50% > LDH/CTF-1-20% > LDH-CTF-1-40%, which proves that LDH/CTF-1-40% has more effective charge separation and electron transfer capabilities.

In summary, all of these results explicitly prove that the combination between LDH and CTF-1 expresses excellent photo-electrochemical properties. However, the photoelectricity performance would decrease when the CTF-1 amount exceeded 40%. The introduction of CTF-1 effectively enhances the visible light absorbability, but excessive CTF-1 could let the heterojunction exhibit more properties of CTF-1, such as lower photocurrent density and higher electrochemical independence (Figure 6c,d), destroying the synergistic effect of the LDH/CTF-1 heterojunction.

### 3.3. Photocatalytic Degradation of TC

**Figure 7:**
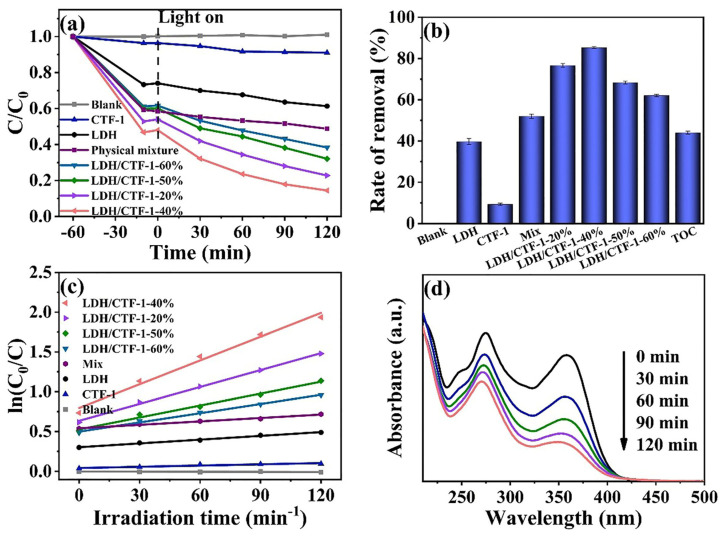
(**a**) Photocatalytic degradation TC curves; (**b**) removal rate of TC solution; (**c**) first-order-kinetic plots of TC degradation; and (**d**) time-dependent UV-vis spectra of TC solution for the LDH/CTF-1-40% sample.

The degradation of TC under visible light irradiation was used to assess the photocatalytic capabilities of LDH, CTF-1, and LDH/CTF-1 nanocomposites. Figure 7a,b show that the blank experiments (without photocatalyst) induced by visible light resulted in little TC degradation as their concentration remained practically constant, indicating that the addition of samples improves photocatalytic TC degradation. After 120 min of illumination, the degradation ratio of TC with LDH/CTF-1-40% was up to 85.6%, while those for LDH, CTF-1, LDH/CTF-1-20%, LDH/CTF-1-50%, LDH/CTF-1-60%, and Mix were 38.7%, 9.0%, 77.2%, 68.0%, 61.6%, and 51.2%, respectively. All of the LDH/CTF-1 heterojunction showed superior photocatalytic behavior to pure LDH and CTF-1 under the same reaction conditions, and the LDH/CTF-1-40% exhibited the best performance, corresponding to the optical characteristic of the samples. The presence of CTF-1 in the nanocomposite was also discovered to have a significant impact on their photocatalytic activity. Figure 7c depicts the kinetics of TC degradation using various photocatalysts. The photocatalytic process agrees well with the first-order reaction kinetics model, and the TC degradation rate constants for several samples were computed appropriately. The degradation rate constant of the LDH/CTF-1-40% nanocomposite was 0.0100 min^−1^, which is about 6.30 times pure LDH and 19.92 times pure CTF-1. Figure 7d shows the TC degradation process with LDH/CTF-1-40% with irradiation time, and the absorption peak ascribed to TC gradually decreases within 120 min of illumination. In addition, after the photocatalytic degradation reaction of TC, the TOC obtained by CTF/LDH-1-40% is 44.58%. Despite all this, the mineralization process of TC contaminant still has no idea.

**Figure 8:**
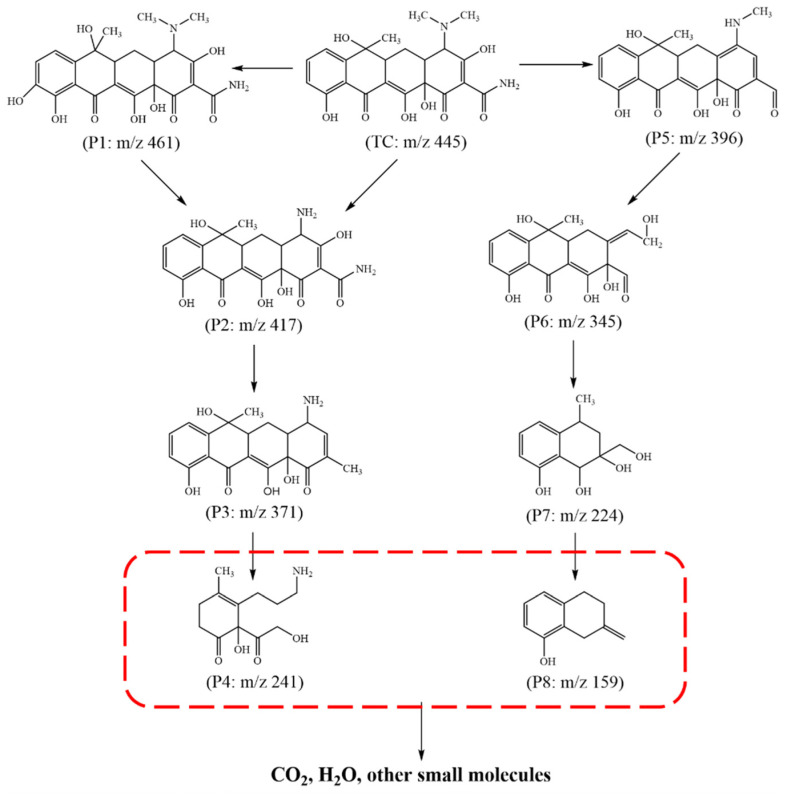
The proposed photocatalytic degradation pathway of TC.

To investigate the TC degradation pathway and mechanism, the original TC solution and intermediates in the process of photocatalytic degradation were analyzed by LC-MS/MS. The most possible photodegradation procedure for the formation of these four key intermediates was provided based on the photodegradation intermediate products. As shown in Figure 8, the molecular ion with m/z = 445 was designated as the TC molecule [41]. Compared to the TC parent molecular, the intermediate with P2 was generated via the loss of two methyl groups owing to the low bond energy of N–C [42,43] and then transformed into the molecular with P3 and P4 by the detachments of amino and H_2_O molecule and cleavage of the fourth ring under the attack of the active species. In addition, the product of P1 was formed by the hydroxylation of TC, which could readily create a phenoxy radical by one-electron oxidation [41,44], further participating in the above reactions. The TC was also converted into the intermediate with P5 produced because of the separation of the N–CH_3_ group and the loss of the H_2_O molecule [35]. After additional oxidation to break the ring and initiate the hydroxylation step, the intermediate containing P6 was formed. Further oxidative breakdown and ring-opening events occurred as the reaction time increased, resulting in intermediates P7 and P8. There are four key degradation reactions for TC throughout the photocatalytic degradation process, including demethylation, ring-opening, decarbonylation, and dihydroxylation [45,46]. These findings show that, after a period of photodegradation time, TC molecules were successfully degraded into some intermediates and small molecules, and the continuing degradation reaction would eventually mineralize CO_2_, H_2_O, and other small molecules.

To further study the removal effect of LDH/CTF-1 on TC, T.E.S.T. software was adopted to analyze the toxicity of TC and its photocatalytic degradation products. As shown in Table S1, the 50% fathead minnow lethal concentration of TC within 96 h is 0.90 mg L^−1^, indicating that TC has strong toxicity to the species. The toxicity of the intermediate produced in the initial stage of photocatalytic reaction may increase slightly, while the lethal concentration of degradation products to minnows was higher than TC as the oxidation process continues, indicating that toxicity was weakened. The 50% lethal concentration of TC and its intermediate products after oral rats were generally high, indicating that there was no lethal harm to the biological species. However, the toxicity of TC could be effectively reduced after photocatalytic decomposition from the overall data changes in the degradation pathway. It is necessary to predict the mutagenicity of TC and its intermediates because of the threat of genetic contamination of TC. According to the photocatalytic degradation pathways of TC corresponding to the data in the table, TC intermediates still have mutagenicity in the early and middle stages of the photocatalytic reaction. However, when intermediates are further mineralized into small fractions such as P4, P7, and P8, they no longer have mutagenicity. In conclusion, the bioavailability of TC can be effectively reduced through the photocatalytic degradation of LDH/CTF-1 heterojunction.

**Figure 9:**
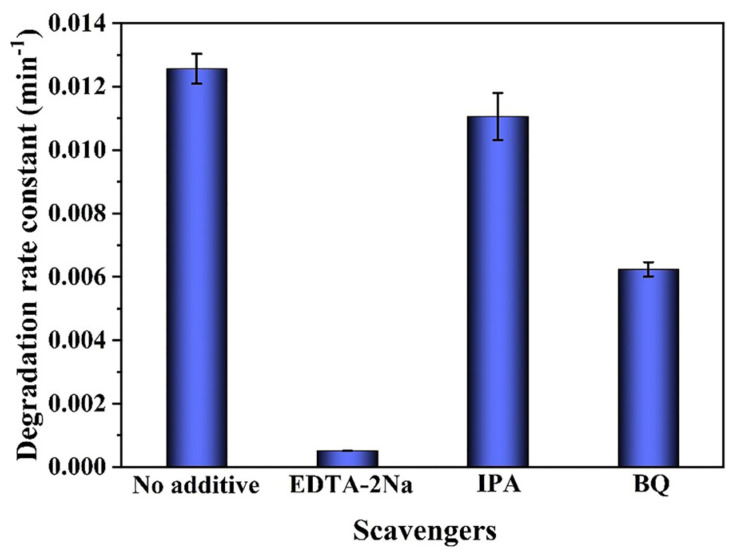
Trapping experiments of the active species for apparent rate constants of TC degradation over the LDH/CTF-1-40% heterojunction.

The oxidation of organic pollutants by h^+^, ·OH, and ·O_2_^−^ radicals is the most common method of photocatalytic degradation [47]. Trapping studies with radical scavengers were carried out to elucidate the photocatalytic mechanism and active species in the photocatalytic degradation of TC on LDH/CTF-1-40% photocatalyst. As the result given in Figure 9, the degradation rate constant of TC could be reached up to 0.013 min^−1^ without radical scavengers. With the addition of EDTA-2Na or IPA scavenger to the TC solution, the degradation rate constant was decreased by about 26 and 2.2 times, respectively, while the degradation rate constant had no obvious change in the presence of BQ. It can be seen from the trapping experiments that h^+^ is the main active substance and the minor role is ·O_2_^−^ in the photocatalytic degradation process.

**Figure 10:**
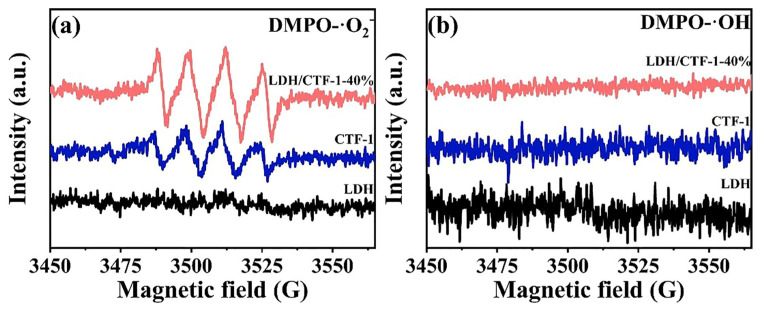
ESR spectra for LDH, CTF-1 and LDH/CTF-1-40% (**a**) DMPO-·O_2_^−^ and (**b**) DMPO-·OH.

Electron spin response (ESR) spin–trap technology was used to further investigate the primary reactive species. The radical trapping compound 5,5-dimethyl-1-pyrroline N-oxide (DMPO) was used to record ESR spectra in the dark and after 10 min of visible light irradiation [48]. As depicted in Figure 10a, no specific spectrum was observed on LDH, and compared with the original CTF-1, the signal intensity of DMPO-·O_2_^−^ on LDH/CTF-1-40% is enhanced. Meanwhile, no DMPO-·OH signal was detected in the pure LDH, CTF-1, and LDH/CTF-1-40% samples (Figure 10b). These results confirm the presence of ·O_2_^−^, while no ·OH could be found during the photocatalytic process. This is consistent with the trapping experimental results.

The stability and reusability of the catalyst are critical factors to consider when evaluating its suitability for practical applications. As shown in Figure S4, the degradation capability of LDH/CTF-1-40% gradually decreased during four consecutive runs. In general, all of the runs exhibited good reusability and more than 65% TC removal efficiency. Furthermore, the XRD spectra of the sample from the beginning to the end of the reaction are unaffected. This study further demonstrated the LDH/CTF-1 heterostructures’ outstanding reusability and stability.

### 3.4. Photocatalytic Mechanism of LDH/CTF-1 Heterostructures

**Figure 11:**
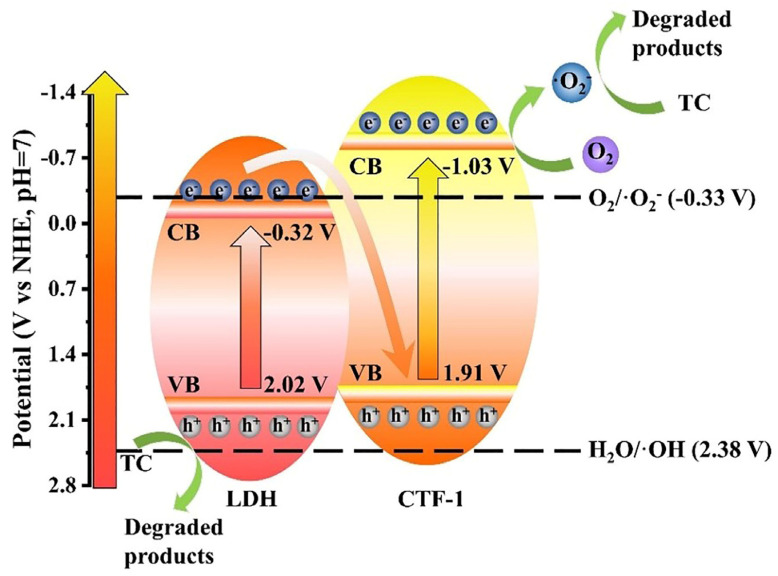
The photocatalytic mechanism for the degradation of TC on Z-Scheme LDH/CTF-1 heterostructures.

In LDH/CTF-1 nanocomposites, the energy levels of LDH and CTF-1 may have a significant impact on charge carrier transfer direction and redox ability. The conduction band (CB) potential was determined using electrochemical Mott–Schottky plots. In comparison to the typical hydrogen electrode (vs. NHE), the values for LDH and CTF-1 were −0.32 and −1.03 V, respectively (Figure S5a,b). The valence band (VB) potential of LDH and CTF-1 vs. SHE should be 2.02 V and 1.91 V, respectively, by their Eg determined in the DRS study. Based on the aforementioned data, the likely transfer behavior of photogenerated carriers and the photocatalytic process involved are hypothesized. The redox potential of O_2_/·O_2_^−^ is −0.33 V at pH = 7, while that of H_2_O/·OH is 2.38 V [49]. If the heterojunction formed between LDH and CTF-1 was classic type-II, the electrons stored in the CB of CTF-1 would transfer into the CB of LDH. Meanwhile, the h^+^ in the VB of LDH would move to the VB of CTF-1 in the same way. Because the CB of LDH was more positive than O_2_/·O_2_^−^, no ·O_2_^−^ would be created. However, these findings contradicted the ESR experiment, which verified the formation of ·O_2_^−^ on LDH/CTF-1-40%. As a result, the type-II charge transfer mechanism for LDH/CTF-1 was shown to be unsuccessful. Using the Z-scheme, we propose a better charge transfer direction for LDH/CTF-1 in this study. The photocatalytic mechanism of Z-scheme LDH/CTF-1 photocatalyst during TC degradation is depicted in Figure 11. The photogenerated electrons in the CB of LDH will recombine with the photogenerated holes in the VB of CTF-1, separating the holes in the VB of LDH and the electrons in the CB of CTF-1. Because of its greater negative redox potential than O_2_/·O_2_^−^, the electrons in the CB of CTF-1 have a potential of −1.03 eV, which can create ·O_2_^−^. While the holes in the VB of LDH have a potential of 2.02 eV, they are unable to oxidize H_2_O to produce ·OH because their redox potential is higher than that of H_2_O/·OH. Both ·O_2_^−^ and h^+^ are active radicals that can continually destroy TC. The internal electric field created by the potent interface between LDH and CTF-1 can speed up the recombination of photogenerated electrons in the CB of LDH and photogenerated holes in the VB of CTF-1. The effective Z-scheme heterostructure preserved with superior oxidic-ability is credited with superior photocatalytic performance.

## 4. Conclusions

In conclusion, an innovative Z-scheme heterogeneous photocatalyst, NiFe-LDH/CTF-1, was fabricated by directly growing NiFe-LDH on CTF-1 for photocatalytic organic pollutants’ degradation. The ability of the as-obtained photocatalysts to degrade TC under visible light was carefully assessed. Compared to pure LDH and CTF-1, the photodegradation of the LDH/CTF-1 heterojunctions for the removal of organic pollutants was superior. The LDH/CTF-1-40% exhibits the highest efficiency of TC degradation (85.6%) and had a degradation rate constant that was approximately 6.30 times and 19.92 times greater than that of pure LDH and CTF-1, respectively. Such astounding activity and stability may be explained by the strongly connected heterointerfaces, increased specific surface area, better light-harvesting capability, and improved charge–carrier dynamics behavior of the semiconductor by the Z-scheme process. This paper demonstrates a successful method for the breakdown of antibiotics that combines organic and inorganic semiconductors with strong photoactivity. The focus of future studies could be on developing efficient regeneration procedures as well as conserving and fixing active components on the heterostructure surface.

## Figures and Tables

**Table 1:** BET surface area and pore volume of LDH, CTF-1, and the LDH/CTF-1-40% samples.

Catalyst	Pore Volume (cm^3^ g^−1^ nm^−1^)	Average Pore Size (nm)	BET Surface Area (m^2^ g^−1^)
LDH	0.95	13	281
CTF-1-40%	0.72	14	207
CTF-1	0.13	24	22

## Data Availability

The data that support the findings of this study are available from the corresponding author upon reasonable request.

## Supplementary Materials

The following supporting information can be downloaded at: https://www.mdpi.com/article/10.3390/nano12234111/s1, Figure S1: XRD patterns of pure LDH, CTF-1, and the CTF-1/LDH-25% sample; Figure S2. EDX elemental mapping images of the LDH/CTF-1-40% nanocomposite; Figure S3. Plots of (αhv)^2^ vs. the energy of absorbed light for pure LDH, CTF-1 and the LDH/CTF-1 nanocomposites; Figure S4. Cycling runs for degradation efficiency of TC (a) and XRD patterns of before and after reaction (b) over the LDH/CTF-1-40%; Figure S5. Mott–Schottky of LDH (a) and CTF-1 (b); Table S1. Toxicity prediction of TC and its intermediates.

## References

[1] Singh R., Singh A.P., Kumar S., Giri B.S., Kim K.-H. (2019). Antibiotic resistance in major rivers in the world: A systematic review on occurrence, emergence, and management strategies. J. Clean. Prod..

[2] Daghrir R., Drogui P. (2013). Tetracycline antibiotics in the environment: A review. Environ. Chem. Lett..

[3] Yang X.R., Chen Z., Zhao W., Liu C.X., Qian X.X., Zhang M., Wei G.Y., Khan E., Ng Y.H., Ok Y.S. (2021). Recent advances in photodegradation of antibiotic residues in water. Chem. Eng. J..

[4] Carvalho I.T., Santos L. (2016). Antibiotics in the aquatic environments: A review of the European scenario. Environ. Int..

[5] Dong H., Zeng G., Tang L., Fan C., Zhang C., He X., He Y. (2015). An overview on limitations of TiO_2_-based particles for photocatalytic degradation of organic pollutants and the corresponding countermeasures. Water Res..

[6] Dong S., Tan Z., Chen Q., Huang G., Wu L., Bi J. (2022). Cobalt quantum dots as electron collectors in ultra-narrow bandgap dioxin linked covalent organic frameworks for boosting photocatalytic solar-to-fuel conversion. J. Colloid Interface Sci..

[7] Xue Y., Tang W., Si C., Lu Q., Guo E., Wei M., Pang Y. (2022). 0D/2D/1D silver-decorated CuPc/Bi_2_MoO_6_ Z-scheme heterojunctions enable better visible-light-driven tetracycline photocatalysis. Opt. Mater..

[8] Jin J., Yu J., Guo D., Cui C., Ho W. (2015). A Hierarchical Z-Scheme CdS–WO3 Photocatalyst with Enhanced CO_2_ Reduction Activity. Small.

[9] Deng S., Li Z., Zhao T., Huang G., Wang J., Bi J. (2022). Direct Z-scheme covalent triazine-based framework/Bi2WO6 heterostructure for efficient photocatalytic degradation of tetracycline: Kinetics, mechanism and toxicity. J. Water Process Eng..

[10] Wu S., Xu Z., Zhang J., Zhu M. (2021). Recent Progress on Metallic Bismuth-Based Photocatalysts: Synthesis, Construction, and Application in Water Purification. Solar RRL.

[11] Gusain R., Gupta K., Joshi P., Khatri O.P. (2019). Adsorptive removal and photocatalytic degradation of organic pollutants using metal oxides and their composites: A comprehensive review. Adv. Colloid Interface Sci..

[12] Ayodhya D., Veerabhadram G. (2018). A review on recent advances in photodegradation of dyes using doped and heterojunction based semiconductor metal sulfide nanostructures for environmental protection. Mater. Today Energy.

[13] Li X., Garlisi C., Guan Q., Anwer S., Al-Ali K., Palmisano G., Zheng L. (2021). A review of material aspects in developing direct Z-scheme photocatalysts. Mater. Today.

[14] Wang L., Zhang Y., Chen L., Xu H., Xiong Y. (2018). 2D Polymers as Emerging Materials for Photocatalytic Overall Water Splitting. Adv. Mater..

[15] Gao Y., Tan Z., Yang R., Huang G., Bi J. (2022). Integrating polyarylether-COFs with TiO_2_ nanofibers for enhanced visible-light-driven CO_2_ reduction in artificial photosynthesis. Appl. Surf. Sci..

[16] Lin G., Sun L., Huang G., Chen Q., Fang S., Bi J., Wu L. (2021). Direct Z-scheme copper cobaltite/covalent triazine-based framework heterojunction for efficient photocatalytic CO_2_ reduction under visible light. Sustain. Energy Fuels.

[17] Zhang G., Zhang X., Meng Y., Pan G., Ni Z., Xia S. (2020). Layered double hydroxides-based photocatalysts and visible-light driven photodegradation of organic pollutants: A review. Chem. Eng. J..

[18] Song B., Zeng Z., Zeng G., Gong J., Xiao R., Ye S., Chen M., Lai C., Xu P., Tang X. (2019). Powerful combination of g-C3N4 and LDHs for enhanced photocatalytic performance: A review of strategy, synthesis, and applications. Adv. Colloid Interface Sci..

[19] Tian J., Zhang J., Xu B., Chen Q., Huang G., Bi J. (2022). An Artificial Photosystem of Metal-Insulator-CTF Nanoarchitectures for Highly Efficient and Selective CO2 Conversion to CO. ChemSusChem.

[20] Bi J., Fang W., Li L., Wang J., Liang S., He Y., Liu M., Wu L. (2015). Covalent Triazine-Based Frameworks as Visible Light Photocatalysts for the Splitting of Water. Macromol. Rapid Commun..

[21] Jiang X., Wang P., Zhao J. (2015). 2D covalent triazine framework: A new class of organic photocatalyst for water splitting. J. Mater. Chem. A.

[22] Tan Z., Zhang P., Chen Q., Fang S., Huang G., Bi J., Wu L. (2021). Visible-light-driven photocatalyst based upon metal-free covalent triazine-based frameworks for enhanced hydrogen production. Catal. Sci. Technol..

[23] Huang G., Niu Q., Zhang J., Huang H., Chen Q., Bi J., Wu L. (2022). Platinum single-atoms anchored covalent triazine framework for efficient photoreduction of CO_2_ to CH_4_. Chem. Eng. J..

[24] Yan J., Zhang X., Zheng W., Lee L.Y.S. (2021). Interface Engineering of a 2D-C3N4/NiFe-LDH Heterostructure for Highly Efficient Photocatalytic Hydrogen Evolution. ACS Appl. Mater. Interfaces.

[25] Xu Y., Hao Y., Zhang G., Lu Z., Han S., Li Y., Sun X. (2015). Room-temperature synthetic NiFe layered double hydroxide with different anions intercalation as an excellent oxygen evolution catalyst. RSC Adv..

[26] Liu M., Jiang K., Ding X., Wang S., Zhang C., Liu J., Zhan Z., Cheng G., Li B., Chen H. (2019). Controlling Monomer Feeding Rate to Achieve Highly Crystalline Covalent Triazine Frameworks. Adv. Mater..

[27] Schwinghammer K., Hug S., Mesch M.B., Senker J., Lotsch B.V. (2015). Phenyl-triazine oligomers for light-driven hydrogen evolution. Energy Environ. Sci..

[28] Yang Y., Li J., Yan T., Zhu R., Yan L., Pei Z. (2020). Adsorption and photocatalytic reduction of aqueous Cr(VI) by Fe3O4-ZnAl-layered double hydroxide/TiO2 composites. J. Colloid Interface Sci..

[29] Tonda S., Kumar S., Bhardwaj M., Yadav P., Ogale S. (2018). g-C3N4/NiAl-LDH 2D/2D Hybrid Heterojunction for High-Performance Photocatalytic Reduction of CO_2_ into Renewable Fuels. ACS Appl. Mater. Interfaces.

[30] Zheng Y., Cheng B., You W., Yu J., Ho W. (2019). 3D hierarchical graphene oxide-NiFe LDH composite with enhanced adsorption affinity to Congo red, methyl orange and Cr(VI) ions. J. Hazard. Mater..

[31] Kuecken S., Acharjya A., Zhi L., Schwarze M., Schomaecker R., Thomas A. (2017). Fast tuning of covalent triazine frameworks for photocatalytic hydrogen evolution. Chem. Commun..

[32] Zhang H., Zhang G., Bi X., Chen X. (2013). Facile assembly of a hierarchical core@shell Fe_3_O_4_@CuMgAl-LDH (layered double hydroxide) magnetic nanocatalyst for the hydroxylation of phenol. J. Mater. Chem. A.

[33] Gultom N.S., Abdullah H., Hsu C.-N., Kuo D.H. (2021). Activating nickel iron layer double hydroxide for alkaline hydrogen evolution reaction and overall water splitting by electrodepositing nickel hydroxide. Chem. Eng. J..

[34] Lin Q., Li L., Liang S., Liu M., Bi J., Wu L. (2015). Efficient synthesis of monolayer carbon nitride 2D nanosheet with tunable concentration and enhanced visible-light photocatalytic activities. Appl. Catal. B Environ..

[35] Kandi D., Behera A., Sahoo S., Parida K. (2020). CdS QDs modified BiOI/Bi_2_MoO_6_ nanocomposite for degradation of quinolone and tetracycline types of antibiotics towards environmental remediation. Sep. Purif. Technol..

[36] Fang P., Wang Z., Wang W. (2019). Enhanced photocatalytic performance of ZnTi-LDHs with morphology control. CrystEngComm.

[37] Zhang S., Zhao Y., Shi R., Zhou C., Waterhouse G.I.N., Wang Z., Weng Y., Zhang T. (2021). Sub-3 nm Ultrafine Cu_2_O for Visible Light Driven Nitrogen Fixation. Angew. Chem. Int. Ed..

[38] Ding X., Liu H., Chen J., Wen M., Li G., An T., Zhao H. (2020). In situ growth of well-aligned Ni-MOF nanosheets on nickel foam for enhanced photocatalytic degradation of typical volatile organic compounds. Nanoscale.

[39] Li F., Sun M., Zhou B., Zhu B., Yan T., Du B., Shao Y. (2022). Z-scheme bismuth-rich bismuth oxide iodide/bismuth oxide bromide hybrids with novel spatial structure: Efficient photocatalytic degradation of phenolic contaminants accelerated by in situ generated redox mediators. J. Colloid Interface Sci..

[40] Li Y., Zhang H., Liu P., Wang D., Li Y., Zhao H. (2013). Cross-Linked g-C_3_N_4_/rGO Nanocomposites with Tunable Band Structure and Enhanced Visible Light Photocatalytic Activity. Small.

[41] Guo F., Huang X., Chen Z., Ren H., Li M., Chen L. (2020). MoS_2_ nanosheets anchored on porous ZnSnO_3_ cubes as an efficient visible-light-driven composite photocatalyst for the degradation of tetracycline and mechanism insight. J. Hazard. Mater..

[42] Zhang Y., Shi J., Xu Z., Chen Y., Song D. (2018). Degradation of tetracycline in a schorl/H_2_O_2_ system: Proposed mechanism and intermediates. Chemosphere.

[43] Xin S., Ma B., Liu G., Ma X., Zhang C., Ma X., Gao M., Xin Y. (2021). Enhanced heterogeneous photo-Fenton-like degradation of tetracycline over CuFeO_2_/biochar catalyst through accelerating electron transfer under visible light. J. Environ. Manag..

[44] Meng F., Ma W., Wang Y., Zhu Z., Chen Z., Lu G. (2020). A tribo-positive Fe@MoS(2)piezocatalyst for the durable degradation of tetracycline: Degradation mechanism and toxicity assessment. Environ. Sci. Nano.

[45] Barhoumi N., Olvera-Vargas H., Oturan N., Huguenot D., Gadri A., Ammar S., Brillas E., Oturan M.A. (2017). Kinetics of oxidative degradation/mineralization pathways of the antibiotic tetracycline by the novel heterogeneous electro-Fenton process with solid catalyst chalcopyrite. Appl. Catal. B Environ..

[46] Jiang D., Wang T., Xu Q., Li D., Meng S., Chen M. (2017). Perovskite oxide ultrathin nanosheets/g-C_3_N_4_ 2D-2D heterojunction photocatalysts with significantly enhanced photocatalytic activity towards the photodegradation of tetracycline. Appl. Catal. B Environ..

[47] Li F., Kang Y., Chen M., Liu G., Lv W., Yao K., Chen P., Huang H. (2016). Photocatalytic degradation and removal mechanism of ibuprofen via monoclinic BiVO_4_ under simulated solar light. Chemosphere.

[48] Huang H., Feng W., Niu Z., Qin X., Liu X., Shan B., Liu Y. (2022). Structural, optical and photocatalytic properties of magnetic recoverable Mn_0.6_Zn_0.4_Fe_2_O_4_@Zn_0.9_Mn_0.1_O heterojunction prepared from waste Mn-Zn batteries. J. Environ. Manag..

[49] Ismael M., Wu Y. (2019). A facile synthesis method for fabrication of LaFeO_3_/g-C_3_N_4_ nanocomposite as efficient visible-light-driven photocatalyst for photodegradation of RhB and 4-CP. New J. Chem..

