# Supplementary material for: Insights into Photocatalytic Degradation Pathways and Mechanism of Tetracycline by an Efficient Z-Scheme NiFe-LDH/CTF-1 Heterojunction
Source: Nanomaterials (Basel). 2022 Nov 22;12(23):4111. doi: 10.3390/nano12234111 (PMC9738193; doi:10.3390/nano12234111)
Supplement: Supplementary file 1 [file nanomaterials-12-04111-s001.zip › nanomaterials-2002074-supplementary.pdf]

nanomaterials

Supplementary Materials

Insights into Photocatalytic Degradation Pathways and Mechanism of Tetracycline by an Efficient Z-Scheme NiFe-LDH/CTF-1 Heterojunction

Jinpeng Zhang [1], Xiaoping Chen [1], Qiaoshan Chen [1], Yunhui He [2], Min Pan [3], Guocheng Huang [1,*] and Jinhong Bi [1,*]

[1] School of Environmental Science and Engineering, Fuzhou University, Fuzhou 350108, China
[2] Fujian College Association Instrumental Analysis Center of Fuzhou University, Fuzhou 350108, China
[3] Department of Applied Science, School of Science and Technology, Hong Kong Metropolitan University, Ho Man Tin, Kowloon, Hong Kong SAR, China
* Correspondence: sysuhgc@gmail.com or huanggch@fzu.edu.cn (G.H.); bijinhong@fzu.edu.cn (J.B.)

1. Methods

1.1. Material and Electrochemical Characterization

Powder X-ray diffraction (PXRD) patterns were obtained on a Bruker D8-advance (Billerica, MA, USA) diffractometer equipped with Cu Kα radiation, operating at a 2θ range from 5° to 80°. Fourier transform infrared (FT-IR) spectra were recorded in the range 4000 to 600 cm-1 by using KBr pellets on a Thermo Scientific Nicolet iS10 spectrometer (Boston, MA, USA). X-ray photoelectron spectroscopy (XPS) measurements were performed on a PHI Quantum 2000 XPS (Washington, WA, USA) system equipped with a monochromatic Al Ka X-ray source. The C 1s peak (284.6 eV) of the surface adventitious carbon was used as the internal reference. Ultra-high resolution scanning electron microscopy (HRSEM) images and Transmission electron microscopy (TEM) images were collected using a Verios G4 UC microscopy (FEI Corp., Hillsboro, OR, USA) and a Tecnai G2 F20 microscope (FEI Corp., Hillsboro, OR, USA), respectively. Nitrogen adsorption and desorption isotherms were carried out at 77 K on an ASAP 2020 apparatus (Micromeritics Instrument Corp., Norcross, GE, USA) and the Brunauer-Emmett-Teller (BET) method was used to calculate the surface area. UV–vis diffuse reflectance spectra (UV-vis DRS) were measured with a Varian Cary 500 UV–vis spectrophotometer (Palo Alto, CA, USA) by using the powder samples with BaSO4 as the reflectance standard. The photoluminescence (PL) spectra were recorded using an Edinburgh FL/FS 900 (Edinburgh, United Kingdom) spectrophotometer with 330 nm excitation light. The electron spin response (ESR) signals of free radicals were examined on a spectrometer (Bruker A300, Billerica, MA, USA) under visible light irradiation ($\lambda > 420$ nm) using 5,5-dimethyl-1-pyrroline N-oxide (DMPO) as a spin-trapped reagent.

The electrochemical properties of the catalysts were determined in a cell with three electrodes on a CHI650E electrochemical workstation. The as-prepared sample, a platinum sheet and Ag/AgCl were applied as the working, reference and counter electrodes, respectively. We fabricated the working electrode through the following process: a 5 mg photocatalyst was immersed in 0.5 mL N, N-dimethylformamide (DMF) and sonicated for 30 min, and then the 10 μL suspension was coated onto fluoride-tin oxide (FTO) glass with a size of 0.5 cm × 0.5 cm. The other area of the FTO glass was smeared with epoxy resin to keep insulated, and then the electrode was dried overnight. The Mott–Schottky and electrochemical impedance spectroscopy (EIS) plots were evaluated through a ZAHNER IM6 in a mixed aqueous solution of KCl (0.1 M), Na_2SO_4 (0.2 M), $K_3[Fe(CN)_6]$ (5 mM) and $K_4[Fe(CN)_6]$ (5 mM). The photocurrent data were recorded on the CHI650E

electrochemical workstation, where the electrolyte and a light source were 0.2 M Na_2SO_4 and a 300 W Xe lamp equipped with a 420 nm filter, respectively.

1.2. Identification of the Degradation Intermediates

The degradation intermediates of TC were identified by a TSQ Quantum Access MAX LC-MS system (Boston, MA, USA) equipped with an Agilent ZORBAX Eclipse Plus C18 (Palo Alto, CA, USA) column (250 × 4.6 mm, 5 μm). The isocratic mobile phase was 0.1% (v/v) of formic acid aqueous solution and acetonitrile (70:30) at a flow rate of 0.8 mL min^{-1}. The injection volume was 10 μL, and the column temperature was 30 °C. UV absorption, at a wavelength of 360 nm, was used for detection. And eluted compounds were detected between m/z 50 and 600.

2. Supplementary Figures

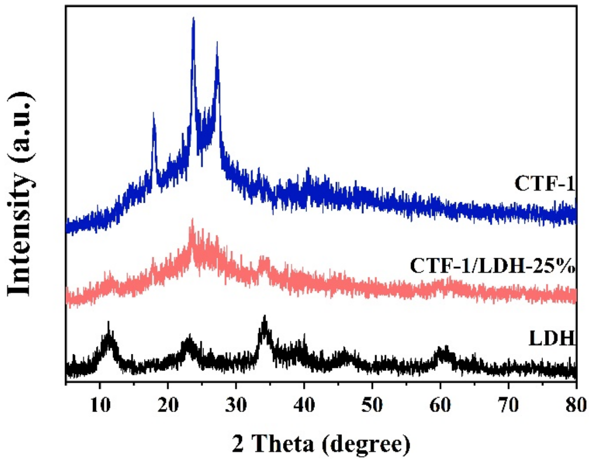

Figure S1. XRD patterns of pure LDH, CTF-1, and the CTF-1/LDH-25%.

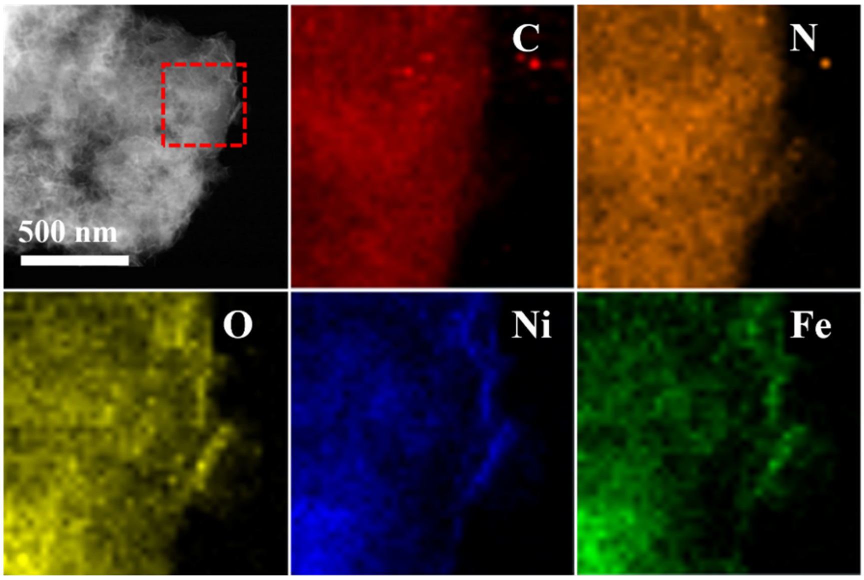

Figure S2. EDX elemental mapping images of the LDH/CTF-1-40% nanocomposite.

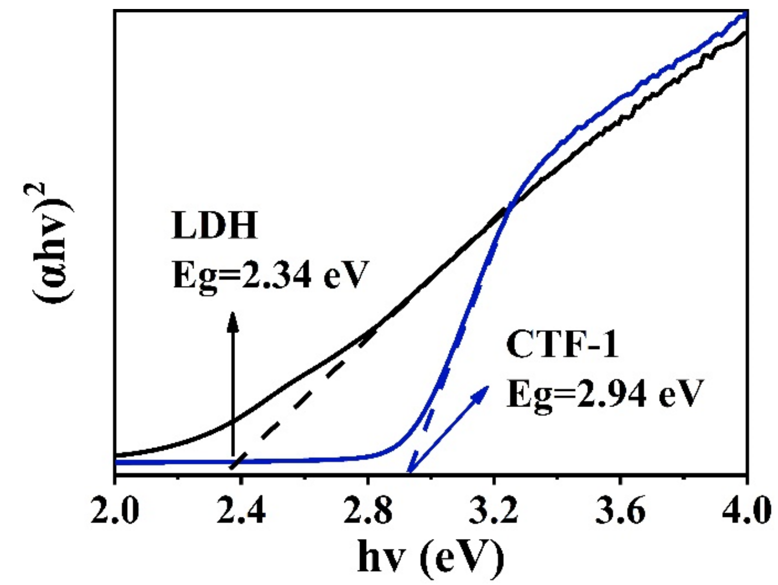

Figure S3. Plots of $(\alpha hv)^2$ vs. the energy of absorbed light for pure LDH, CTF-1 and the LDH/CTF-1 nanocomposites.

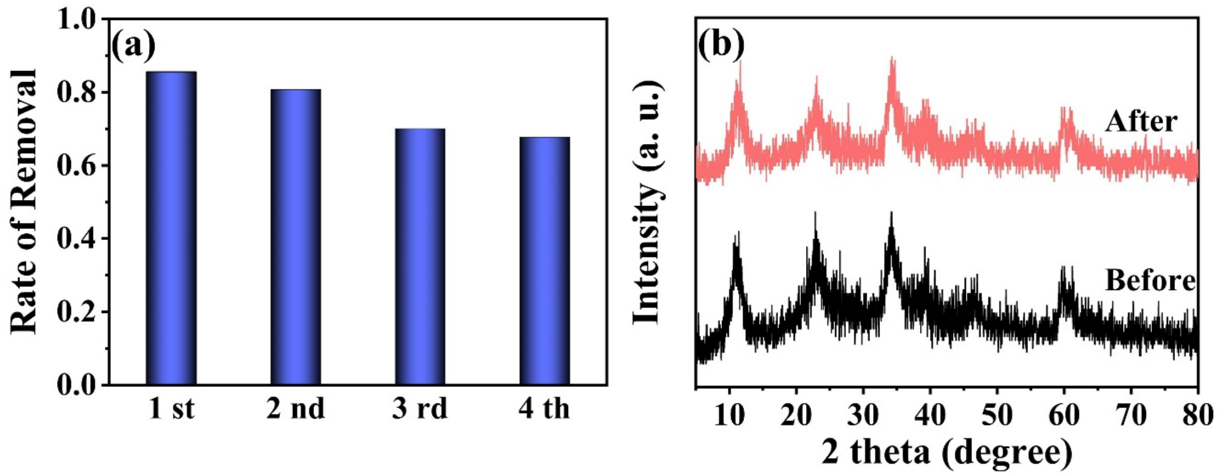

Figure S4. Cycling runs for degradation efficiency of TC (**a**) and XRD patterns of before and after reaction (**b**) over the LDH/CTF-1-40%.

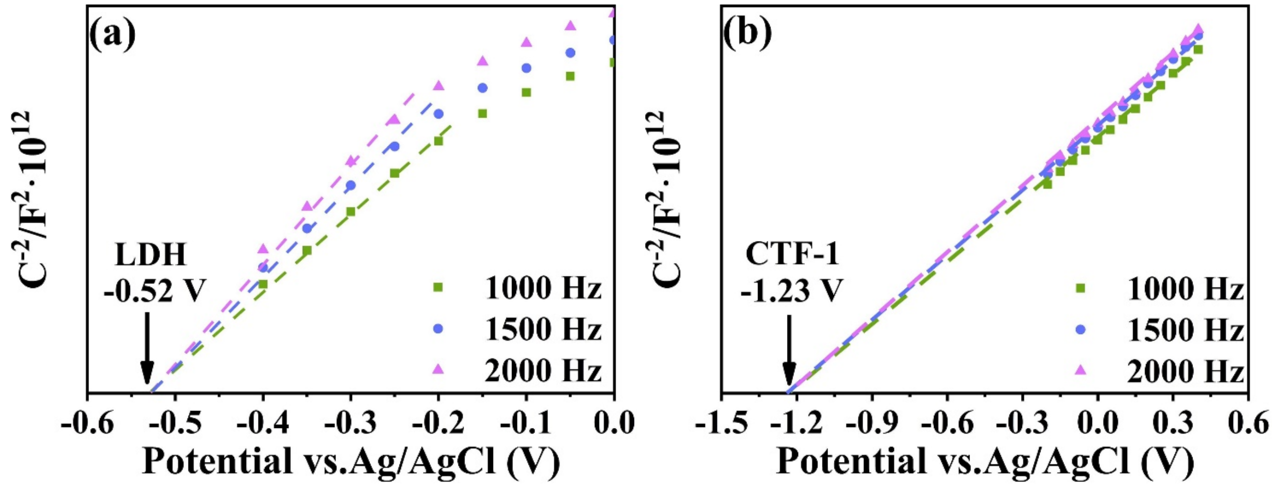

Figure S5. Mott-Schottky of LDH (**a**) and CTF-1 (**b**).

Table S1. Toxicity prediction of TC and its intermediates.

**Toxic endpoint**	**Fathead minnow LC_{50}-96 h (mg L^{-1})**	**Oral rat LD_{50} (mg L^{-1})**	**Mutagenicity**
**TC**	0.90	1068.64	0.60 (positive)
**P1**	0.79 (−)	1615.37 (+)	0.64 (positive)
**P2**	0.47 (−)	1568.39 (+)	0.65 (positive)
**P3**	2.75 (+)	1317.21 (+)	0.81 (positive)
**P4**	603.22 (+)	1656.29 (+)	0.23 (negative)
**P5**	0.85 (+)	1029.30 (−)	0.65 (positive)
**P6**	3.36 (+)	NA	0.91 (positive)
**P7**	90.24 (+)	3679.6 (+)	0.13 (negative)
**P8**	3.74 (+)	NA	−0.03 (negative)
